# The Myelin and Lymphocyte Protein MAL Is Required for Binding and Activity of *Clostridium perfringens* ε-Toxin

**DOI:** 10.1371/journal.ppat.1004896

**Published:** 2015-05-20

**Authors:** Kareem Rashid Rumah, Yinghua Ma, Jennifer R. Linden, Myat Lin Oo, Josef Anrather, Nicole Schaeren-Wiemers, Miguel A. Alonso, Vincent A. Fischetti, Mark S. McClain, Timothy Vartanian

**Affiliations:** 1 Brain and Mind Research Institute, Weill Cornell Medical College, New York City, New York, United States of America; 2 Laboratory of Bacterial Pathogenesis and Immunology, The Rockefeller University, New York City, New York, United States of America; 3 Neurobiology, Department of Biomedicine, University Hospital Basel, University of Basel, Basel, Switzerland; 4 Centro de Biología Molecular Severo Ochoa, CSIC-UAM, Cantoblanco, Madrid, Spain; 5 Department of Medicine, Division of Infectious Diseases, Vanderbilt University School of Medicine, Nashville, Tennessee, United States of America; University of Illinois, UNITED STATES

## Abstract

*Clostridium perfringens* ε-toxin (ETX) is a potent pore-forming toxin responsible for a central nervous system (CNS) disease in ruminant animals with characteristics of blood-brain barrier (BBB) dysfunction and white matter injury. ETX has been proposed as a potential causative agent for Multiple Sclerosis (MS), a human disease that begins with BBB breakdown and injury to myelin forming cells of the CNS. The receptor for ETX is unknown. Here we show that both binding of ETX to mammalian cells and cytotoxicity requires the tetraspan proteolipid Myelin and Lymphocyte protein (MAL). While native Chinese Hamster Ovary (CHO) cells are resistant to ETX, exogenous expression of MAL in CHO cells confers both ETX binding and susceptibility to ETX-mediated cell death. Cells expressing rat MAL are ~100 times more sensitive to ETX than cells expressing similar levels of human MAL. Insertion of the FLAG sequence into the second extracellular loop of MAL abolishes ETX binding and cytotoxicity. ETX is known to bind specifically and with high affinity to intestinal epithelium, renal tubules, brain endothelial cells and myelin. We identify specific binding of ETX to these structures and additionally show binding to retinal microvasculature and the squamous epithelial cells of the sclera in wild-type mice. In contrast, there is a complete absence of ETX binding to tissues from MAL knockout (MAL-/-) mice. Furthermore, MAL-/- mice exhibit complete resistance to ETX at doses in excess of 1000 times the symptomatic dose for wild-type mice. We conclude that MAL is required for both ETX binding and cytotoxicity.

## Author Summary

*Clostridium perfringens* epsilon-toxin is a potent pore-forming toxin responsible for a devastating central nervous system disease in livestock and has been suggested as a possible environmental trigger for Multiple Sclerosis. Epsilon-toxin binds with great specificity to a restricted number of host cell types and structures, for example gut epithelial cells, blood-brain barrier endothelial cells, and myelin. While most pore-forming toxins achieve binding through specific interaction with respective receptors on the cell membrane, the receptor for epsilon-toxin, however, is unknown. In this report we identify the Myelin and Lymphocyte protein, MAL, as being necessary for binding and cytotoxic effects of epsilon-toxin, and we show its second extracellular loop is critical in this novel function. At a physiological level, mice homozygous for a targeted deletion of the MAL gene lack sensitivity to epsilon-toxin whereas the toxin is lethal in wild-type mice. These observations lead to the possibility that MAL is a candidate receptor for epsilon-toxin. However, we have not demonstrated a physical interaction between epsilon-toxin and MAL.

## Introduction

*Clostridium perfringens* is a gram-positive, spore-forming, anaerobic bacillus that is possibly the most widespread pathogenic bacterium in the world [1, 2]. Conventionally, the species is categorized into five toxinotypes, A-E, based on carriage of one or more of the major toxin genes (alpha, beta, epsilon, or iota). *C*. *perfringens* types B and D carry the epsilon toxin (ETX) gene [1, 2]. In all, the *C*. *perfringens* species produces a remarkable seventeen exotoxins, and of these, ETX is by far the most deadly, ranked the third most potent *Clostridial* toxin following botulinum and tetanus toxins [3]

The ETX-producing *C*. *perfringens* type B and D strains are less prevalent than the type A strain and are best studied in ruminant livestock where colonized animals are susceptible to a devastating enterotoxemia disease, which is characterized by loss of vision, paresis, ataxia and other signs of CNS dysfunction [4–7]. *C*. *perfringens* type B or type D can be readily isolated from cultures of animals with characteristic signs of ETX enterotoxemia [8–10].

In *C*. *perfringens* colonized animals, ETX is typically secreted into the intestinal lumen as a 32.9 kDa protoxin (pro-ETX) and enzymatically activated into a 29 kDa toxin by extracellular trypsin, chymotrypsin, or a *C*. *perfringens* encoded lambda protease [11–15]. However, some strains may be able to activate ETX intracellularly [16]. Activated ETX is then absorbed into the bloodstream through partially understood mechanisms [17]. ETX demonstrates a unique tropism for kidney [18], CNS endothelial cells, and brain [19, 20]. Within the bloodstream, ETX encounters the CNS endothelium, causes disruption of BBB, and allows entry of toxin into the brain [21–25]. Once in the brain, ETX binds with high affinity to white matter and this is presumably responsible for its well-described effect of causing white matter lesions in sheep, goats and mice[6, 19, 26–30]. In light of these observations, it has been hypothesized that there may be a connection between ETX and MS [31]. Having obtained the first evidence supporting this notion, we recently proposed that ETX is an environmental factor responsible for initiating lesion formation in MS [32].

Despite its potency and unique tropism for CNS tissues, the mammalian receptor for ETX remains unidentified. Based on prior investigation, it has been postulated that the ETX receptor is a glycoprotein residing in detergent resistant membranes or lipid rafts [33–36]. ETX has also been reported to bind to the extracellular domains of Hepatitis A Virus Receptor 1 (HAVCR1) immobilized on paramagnetic beads [37]. However, although knockdown of HAVCR1 reduces ETX toxicity in previously susceptible cells [37], transfection of ETX-resistant cells with HAVCR1 fails to confer ETX binding and/or susceptibility. These experiments suggest HAVCR1 contributes to ETX toxicity but is insufficient to confer ETX toxicity. Since ETX specifically binds to distal collecting duct epithelium of the kidney [18, 38] and to myelin in the CNS [19, 23, 39, 40], and since the putative ETX receptor is thought to localize to lipid rafts, we searched the National Library of Medicine databases for proteins residing within lipid rafts of distal renal epithelium and myelin. The tetraspan proteolipid MAL [41, 42] uniquely fits these specific requirements. Therefore, we sought to test the hypothesis that MAL is the ETX receptor.

The crystal structure of active ETX reveals an elongated structure with three principle domains sharing structural and conformational homology with the aerolysin family of pore-forming toxins[43]. Domain 1 contains the putative receptor-binding site and includes a cluster of aromatic residues[44] within the N-terminus. An amphipathic beta-hairpin within domain 2 contributes to ETX insertion into the plasma membrane and channel architecture. Domain 3 is thought to be involved in protein-protein interactions between monomers that stabilize oligomers. The receptor-binding domain is distinct from the domain(s) required for oligomerization. Pro-ETX binds to receptive cells with the same affinity as active toxin but does not integrate into the membrane to generate pore-forming oligomers. Formation of the heptameric pore requires cleavage of the carboxy-terminal 22 amino acids [45, 46]. Upon binding to susceptible mammalian cells, ETX monomers form a heptameric prepore [47], which inserts into the plasma membrane generating a stable asymmetric pore of ~500 Da exclusion [48].

MAL is a highly hydrophobic, tetraspan membrane proteolipid with two extracellular loops. It is found in particular polarized epithelial cells [49], oligodendrocytes [42, 50, 51] and human T lymphocytes [52]. In human T cells, MAL is expressed on the plasma membrane and is implicated in the formation of immunological synapses [53, 54]. In the nervous system, MAL is expressed by oligodendrocytes and Schwann cells and localizes to compact as well as non-compact myelin membranes, and is thought to play a role in myelin biogenesis and function [55]. In polarized epithelial cells, MAL localizes to the apical membrane and is thought to shuttle between the Trans-Golgi-Network (TGN) apparatus and the plasma membrane, sorting apically associated membrane proteins to the surface [56, 57]. Moreover, MAL plays critical roles in the formation, stabilization and maintenance of lipid rafts [54, 55, 57–64]. Through a combination of gain and loss of function experiments we show that MAL is required for binding and activity of ETX.

## Results

**Fig 1:**
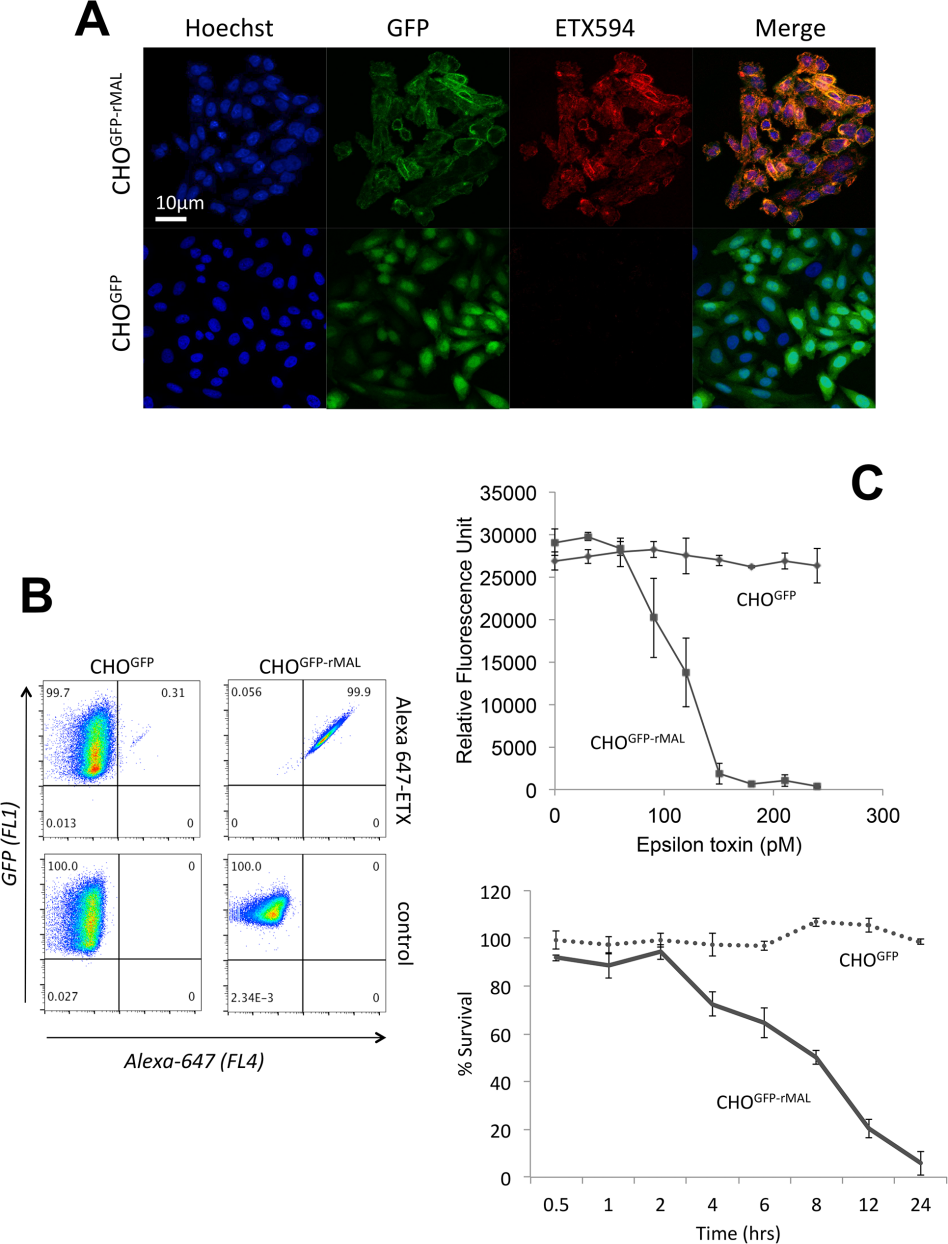
MAL expression in CHO cells is required to confer specific binding of ETX. Stable clones of CHO cells expressing either GFP (CHO^GFP^) or GFP-rMAL (CHO^GFP-rMAL^) were assessed for binding with Alexa-594 labeled ETX (ETX594). (**A**) Confocal images of fixed CHO^GFP^ or CHO^GFP-MAL^ cells treated with 50 nM ETX594 for 60 minutes. Cells were washed and counter-stained with Hoechst (blue) to identify nuclei. There is essentially complete overlap of ETX594 (red) and GFP-MAL (green) signals in cells expressing GFP-MAL. Note that GFP-MAL predominantly localizes to the plasma membrane. In contrast, there is no detectable ETX-594 signal in the cells expressing GFP, which is localized to the cytoplasm. (**B**) Flow cytometry analysis shows scatter plots of living CHO^GFP^ or CHO^GFP-MAL^ cells incubated with 20 nM Alexa-647-ETX. Double positives (GFP and Alexa-647) are observed for CHO^GFP-MAL^ but not CHO^GFP^ cells incubated with Alexa 647-ETX. Thus in both fixed (A) and live cells (B), MAL expression results in specific binding of ETX to the cell surface. (**C**) ETX induces dose- and time-dependent cell death in MAL expressing cells. Cell viability was assessed using PrestoBlue as an indicator of metabolically active cells. CHO^GFP-rMAL^ expressing cells exhibit ETX sensitivity in a dose (upper panel) and time (lower panel) dependent fashion. CHO^GFP^ cells fail to exhibit ETX sensitivity at any of the administered doses or time points. Results were normalized to the fluorescent signal from cells incubated in the absence of toxin (100%) and in 0.1% Triton X-100 (0%). Data shown are means and SD from a single experiment and are representative of four independent triplicate experiments.

Specific binding of active ETX to its receptor is necessary for prepore assembly since resistant cells fail to form heptamers even at high concentrations of ETX [47]. If MAL is required for binding of ETX to cells, then expression of MAL in ETX-resistant cells should confer binding of the toxin and render the cells susceptible to ETX-mediated cell death. To test this hypothesis, we stably expressed a green fluorescent protein (GFP)-rat MAL fusion protein (GFP-rMAL) construct in ETX resistant Chinese Hamster Ovary (CHO) cells (CHO^GFP-rMAL^), and identified that these cells were able to bind Alexa-594 labeled ETX, while CHO cells transfected with GFP alone (CHO^GFP^) did not bind the toxin (**Fig 1A**). Alexa-594 labeled ETX retained normal function in cytotoxicity assays (**S1 Fig**). To confirm and quantify binding of ETX to MAL expressing cells, we tested these two stably transfected cell lines by flow cytometry using Alexa-647 labeled ETX (laser 640nm/detector 675 nm). ETX showed robust binding to CHO^GFP-rMAL^ cells, while failing to bind to CHO^GFP^ controls (**Fig 1B**). To assess the effects of ETX on cell viability in CHO^GFP-rMAL^ and CHO^GFP^ cells, we designed an assay using PrestoBlue as a cell viability indicator. PrestoBlue is a resazurin-based cell permeable compound converted to the fluorescently active resorufin in the reducing environment of metabolically active cells [65–67]. ETX killed CHO^GFP-rMAL^ cells in a dose- and time-dependent fashion (**Fig 1C**). In contrast, CHO^GFP^ cells remained completely viable at all doses of ETX tested. In addition, we assessed cell death using propidium iodide (PI) exclusion as an independent assay for cell death [68, 69]. CHO^GFP^ or CHO^GFP-rMAL^ cells were treated with 500 pM ETX 500pM and monitored for PI uptake over a 24-hour period. By the 4-hour time point, we observed robust PI uptake in the CHO^GFP-rMAL^ but not the CHO^GFP^ control cells (**S2 Fig**). By contrast, after 24 hours, PI uptake remained undetectable in the CHO^GFP^ controls. Taken together, these experiments support the conclusion that MAL expression in ETX resistant cells is required to impart both binding and sensitivity to toxin.

**Fig 2:**
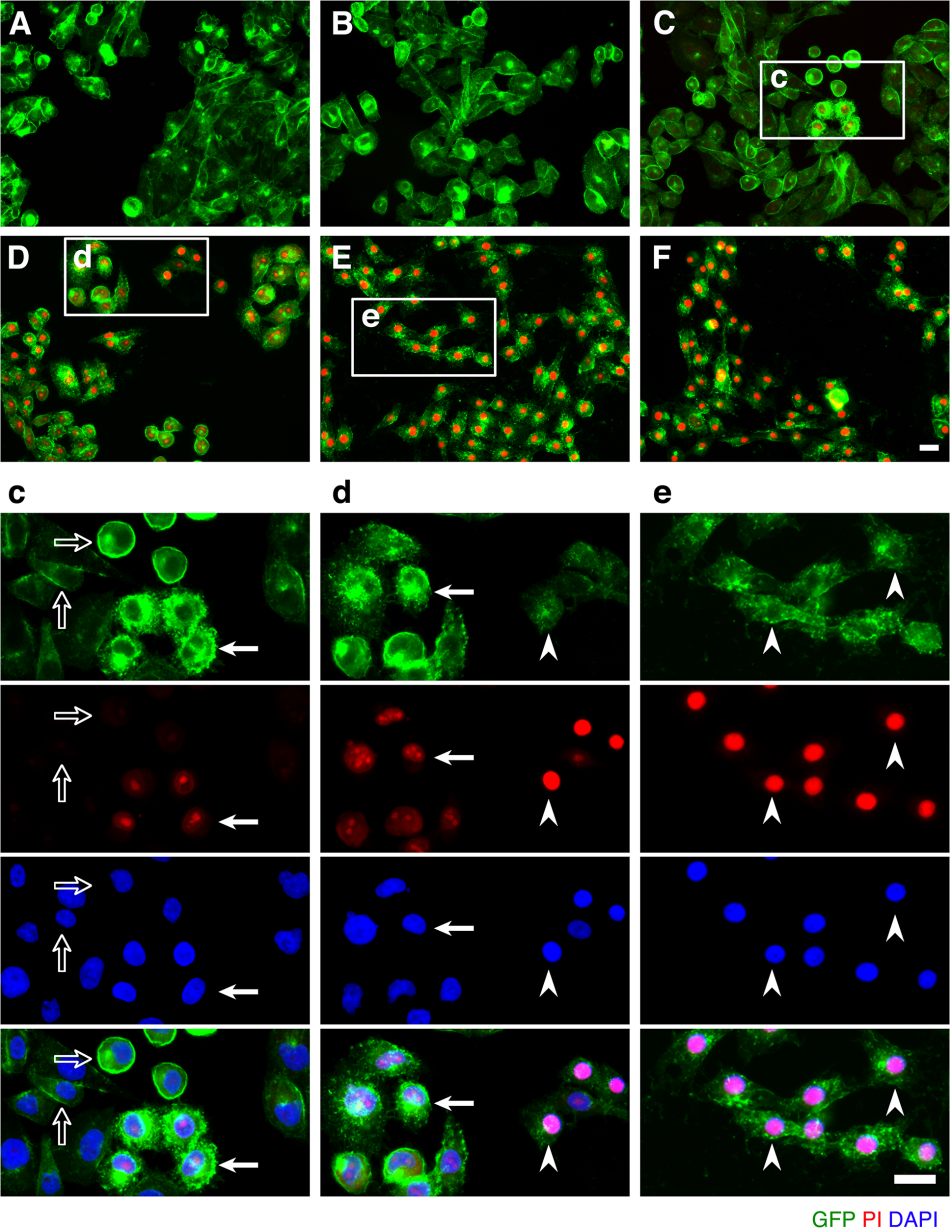
ETX induces rapid internalization of rMAL that precedes or coincides with cell death. CHO cells stably expressing GFP-rMAL (green) were treated with 50 nM ETX in the presence of 2 μg/ml PI (red) and fixed at 0 minutes (**A**), 5 minutes (**B**), 15 minutes (**C**), 30 minutes (**D**), 60 minutes (**E**), and 90 minutes (**F**) following the treatment. Regions (**c**-**e**) framed in C-E are shown at a higher magnification to illustrate morphological details. Images in c-e are shown in separate fluorescence channels and as an overlay plus a DAPI-counterstain. Hollow arrows in c point to the cells on which MAL is largely present on the cell surface. Noticeably, these cells are negative for PI staining. The morphological heterogeneities likely reflect different phases of cell cycle in individual cells within the population. Arrows in c and d point to the cells displaying intracellular vesicles of MAL protein, which indicate an ongoing MAL internalization process in response to ETX. These are dying cells that exhibit PI inclusion in their nuclei with a higher staining density in nucleoli. Arrowheads in d and e point to the dead cells that display condensed nuclei brightly stained with PI and contain intracellular MAL protein. Scale bars represent 20 μm. Data shown are representative of at least three independent experiments.

We next sought to determine the temporal relationship between ETX pore formation and morphologic indicators of cell death. To accomplish this, we incubated CHO^GFP-rMAL^ cells with 50 nM ETX for increasing times and assessed both PI uptake and nuclear morphology. PI has an estimated van der Waals volume of 447 cubic Å. The size of the propidium cation limits passage of PI through membrane pores, and the van der Waals dimensions of propidium requires a minimum diameter of 1.5 nm for a propidium-permeable pore [70]. Studies examining the size and physical properties of the pores formed by ETX suggest an asymmetrical shape of the pore with its larger radius at 1.0 nm [69, 71]. Therefore, the ETX heptameric pore is likely permeable to PI. It has been previously shown that ETX induces permeability of MDCK cells to PI, and that small molecule antagonists that do not block heptamerization but do block the pore, inhibit PI entry into cells [66, 68, 72]. In addition to time-course, we examined the change of localization patterns of MAL in response to ETX exposure. In untreated CHO^GFP-rMAL^ cells and for those treated with ETX for 5 minutes, MAL is localized primarily in the plasma membrane with some perinuclear staining (**Fig 2A** and **2B**). As shown, the complete exclusion of PI indicates preserved membrane integrity in these cells. At 15 minutes of ETX treatment some cells retain normal appearing plasma membrane morphology while others reveal disruption of the plasma membrane and internalization of MAL into vesicles. Interestingly, those cells with normal MAL localization on the plasma membrane completely exclude PI whereas cells with disrupted membrane architecture and internalization of MAL are PI-positive (**Fig 2C**). Cells that have incorporated PI show intense localization of PI in nucleoli and faint staining of morphologically normal nuclei. By 30 minutes of treatment, the majority of CHO^GFP-rMAL^ cells shows disruption of the integrity of the plasma membrane and incorporation of PI (**Fig 2D**). However, incorporation of PI at 30 minutes of treatment falls into one of two morphologic categories: intense staining of nucleoli with faint staining of morphologically normal nuclei, or intense staining of condensed pyknotic nuclei characteristic of apoptosis. By 60 minutes of ETX treatment, all the cells show disrupted plasma membranes and all of the PI staining is in pyknotic condensed nuclei (**Fig 2E**).

**Fig 3:**
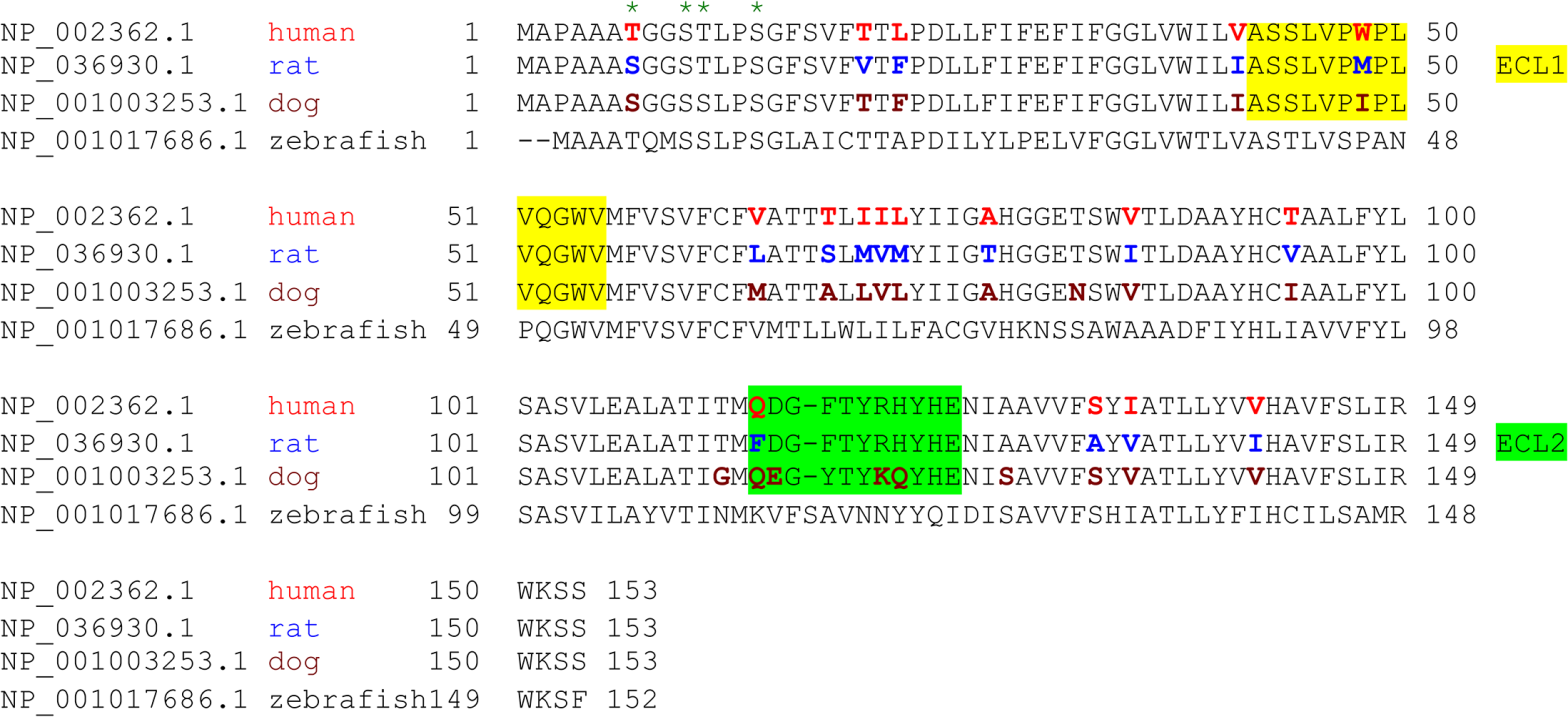
Species variation in the amino acid sequence of MAL. Alignment analysis of human, rat, dog, and zebrafish MAL amino acid (aa) sequences was performed with the multiple sequence alignment program ClustalW2 [100]. The sequence corresponding to the extracellular loop 1 (ECL1) and the extracellular loop 2 (ECL2) is highlighted in yellow and green, respectively. The aa residues differed among human (red), rat (blue), and dog (brown) are color-marked.

**Fig 4:**
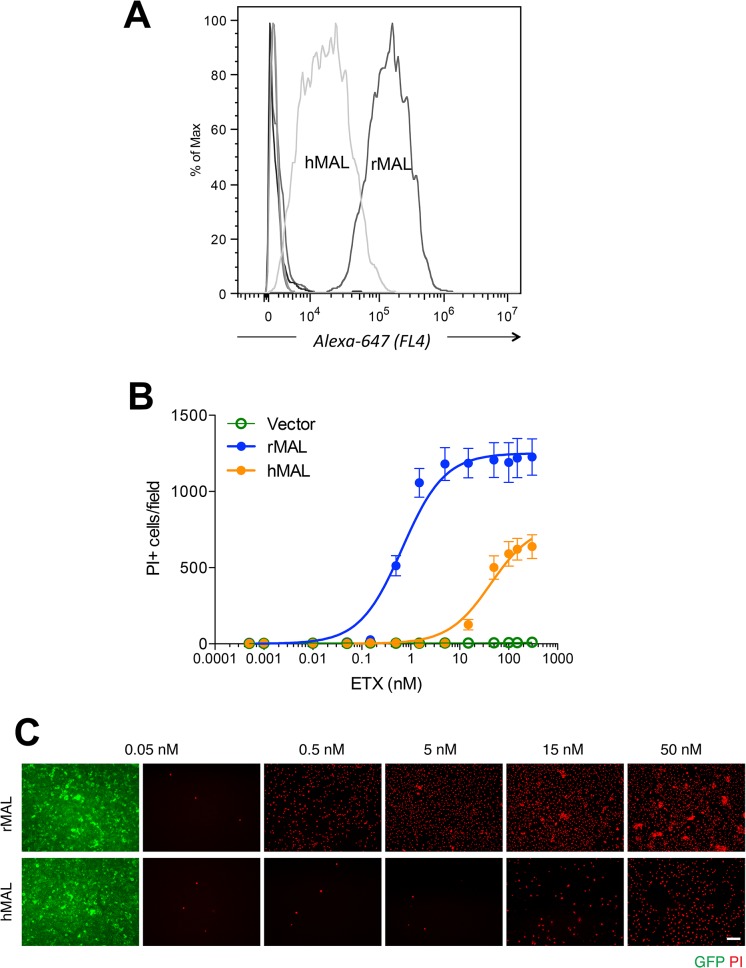
Differential binding and cytotoxicity of ETX to cells expressing MAL orthologues. (**A**) FACS histogram of Alexa 647-labeled ETX binding to cells transiently transfected with MAL expression vectors. As shown, ETX binds to cells expressing rMAL with greater affinity than to hMAL, whereas it fails to bind to CHO cells transiently transfected with constructs expressing zebrafish MAL, hMAL-L2F, or the GFP vector alone (overlapping curves at far left of the plot). (**B**) CHO cells stably expressing GFP-alone (vector), GFP-rMAL (rMAL), or GFP-hMAL (hMAL) were treated with ETX at concentrations ranging from 0 to 300 nM in the presence of 2 μg/ml PI (red) for 6 hours and live-imaged for assessing cell death. (**C**) Representative images show the ETX effect in GFP-rMAL (green) or GFP-hMAL-expressing CHO cells at critical concentrations. Scale bar represents 100 μm. Data are from a single experiment and are representative of four independent triplicate experiments. Data shown in B are means and SD.

If MAL is the receptor or a direct biding partner for ETX, then the protein-protein interaction is most likely to occur between the receptor binding domain of ETX and the extracellular domain(s) of MAL. The primary structure of full-length MAL predicts a tetraspan integral membrane protein with two extracellular loops, ECL1 and ECL2 [41, 51]. MAL shares 87–89% of amino acid residue identity among human, rat, and dog (**Fig 3**). Sequence alignment highlighting ECL1 and ECL2 for rat and human MAL reveals a single amino acid substitution in each of the two domains (**Fig 3**). In contrast, zebrafish MAL shares significantly less amino acid identity with either human or rat MAL, especially within ECL2. To determine if sequence differences in MAL orthologues influence ETX binding and cytotoxicity, we first transiently transfected CHO cells with vectors expressing human, rat, or zebrafish MAL and analyzed binding by flow cytometry. ETX fails to bind to cells expressing either zebrafish MAL or GFP control (**Fig 4A**). ETX binds to both human and rat MAL expressing cells but displays an approximate ten-fold increase in binding affinity for rat MAL when compared to the human orthologue (**Fig 4A**). We then generated stable CHO cells lines expressing rMAL, hMAL, or the GFP vector alone and tested these cells for sensitivity to ETX. Cells were treated with escalating concentrations of ETX for 6 hours followed by live-imaging in the presence of PI to assess pore formation and cell death. As shown, rMAL expressing CHO cells are markedly more sensitive to ETX than those expressing hMAL (**Fig 4B**). The IC50 for ETX-induced cell death was 0.6 nM for rMAL and 40 nM for hMAL. Notably, after 6 hours of treatment, rMAL-expressing CHO cells show evidence of cell death at 0.5 and 5.0 nM ETX, whereas those expressing hMAL are still PI- negative (**Fig 4C**).

**Fig 5:**
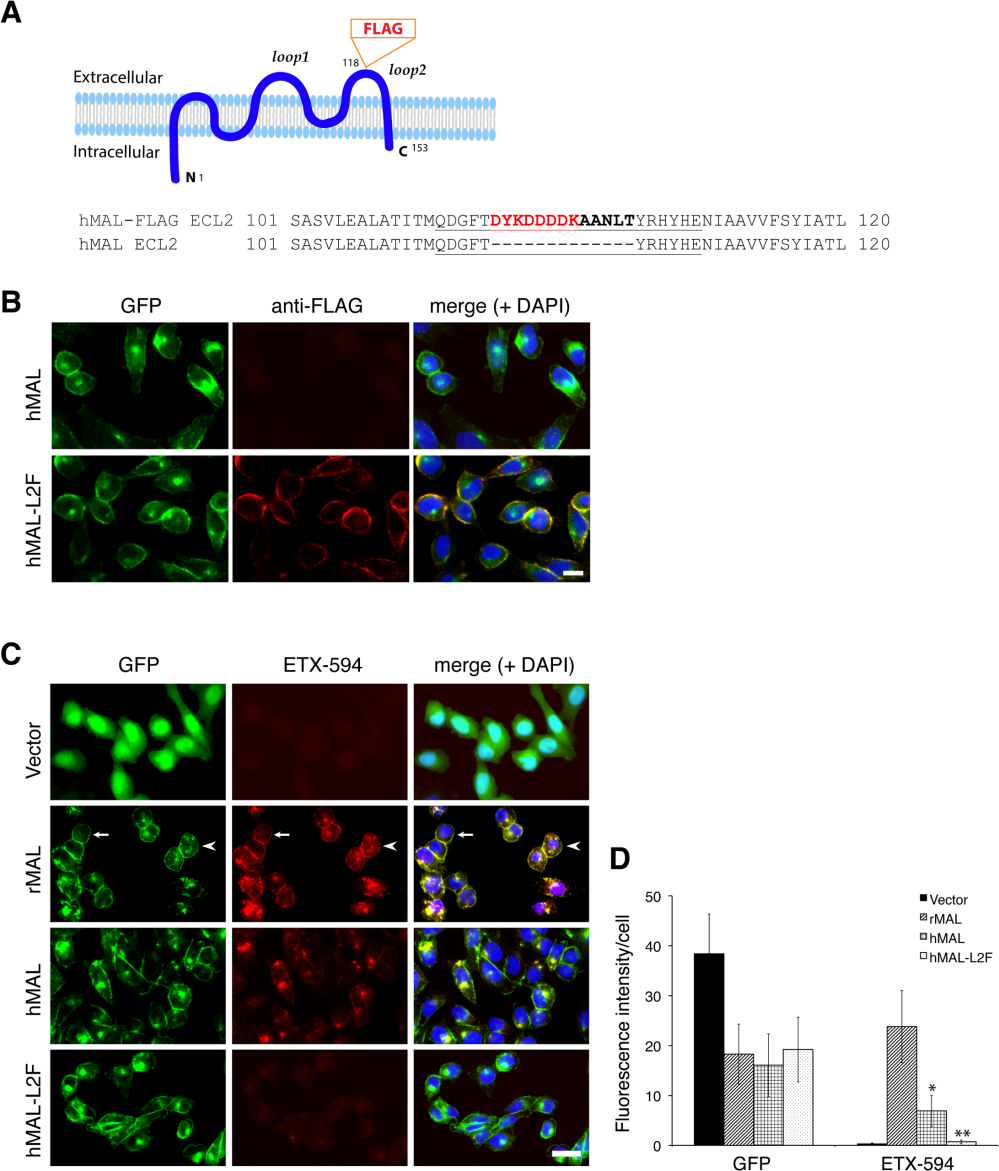
ETX binding requires the second extracellular loop of MAL. (**A**) Generation of cells stably expressing either hMAL or hMAL-GFP harboring a FLAG insertion in its second extracellular loop (hMAL-L2F) fused with N-terminal GFP protein. (**A**) Schematic illustration of hMAL structure (upper panel) and insertion of a 15 aa-sequence, which contains the FLAG epitope (DYKDDDDK) marked in red and a spacer sequence in dark bold (lower panel), in the second extracellular loop (loop 2 or ECL 2) in hMAL. Amino acid residues within loop 2 are underlined. The diagram is adapted with modifications from our previous publication [56]. (**B**) Confirmation of the FLAG tag in hMAL-L2F-expressing CHO cells by anti-FLAG antibody live staining. Scale bar represents 10 μm. (**C** and **D**) Representative images (C) and statistical analysis (D) show that expression of MAL is required for ETX binding, whereas insertion of a FLAG segment in loop 2 of hMAL eliminates ETX binding. CHO cells stably expressing GFP-alone (vector), GFP-rMAL (rMAL), GFP-hMAL (hMAL), or GFP-hMAL-L2F (hMAL-L2F) (green) were incubated with 25 nM Alexa 594-conjugated ETX (ETX-594, red) for 1 hour before fixation for fluorescence analysis. In the second row images, arrows indicate cells on which MAL distribution and ETX-594 labeling occur largely on the cell surface; arrowheads indicate cells undergoing MAL internalization, in which ETX-594 co-localizes with MAL on both the cell membrane and intracellular vesicles; asterisk indicate cells of which the surface representation of MAL and ETX-594 is lost. Binding of ETX is quantitatively greater for cells expressing rMAL compared to hMAL. Scale bar represent 20 μm. Data are from a single experiment and are representative of three (B) or four (C and D) independent triplicate experiments. Data shown in D are means and SD.

To determine if disruption of the primary structure within ECL1 or ECL2 impacts ETX binding to MAL, we generated stable cell lines expressing mutants of the human MAL harboring the FLAG epitope in ECL1 or ECL2 [56], respectively designated hMAL-L1F and hMAL-L2F (**Fig 5A**). We assessed targeting and orientation of the mutant proteins by fluorescence microscopy for both GFP and the FLAG epitope. Stable clones of hMAL-L1F-expressing CHO cells were generated but these failed to reliably target to the plasma membrane and were therefore not used in subsequent analyses. hMAL and hMAL-L2F are targeted to the plasma membrane as well as show some peri-nuclear localization in both live (**Fig 5B)** and fixed cells (**Fig 5C**). In live cultures the anti-FLAG antibody binds to hMAL-L2F expressing cells (**Fig 5B**). This indicates extracellular localization of the FLAG epitope and thus of ECL2, which is consistent with previous description [56, 60]. We then assessed binding and localization of Alexa-594 labeled ETX to cells expressing rMAL, hMAL, hMAL-L2F, and GFP control. Expression levels and subcellular localization patterns for all of three fusion proteins (rMAL, hMAL, and hMAL-L2F) are similar, as shown by GFP fluorescence intensity and distribution (**Fig 5C**). ETX binding is greater in magnitude for rMAL than for hMAL (**Fig 5D**). ETX completely fails to bind to cells expressing hMAL-L2F, underscoring the potentially important role of this domain in ligand interaction.

**Fig 6:**
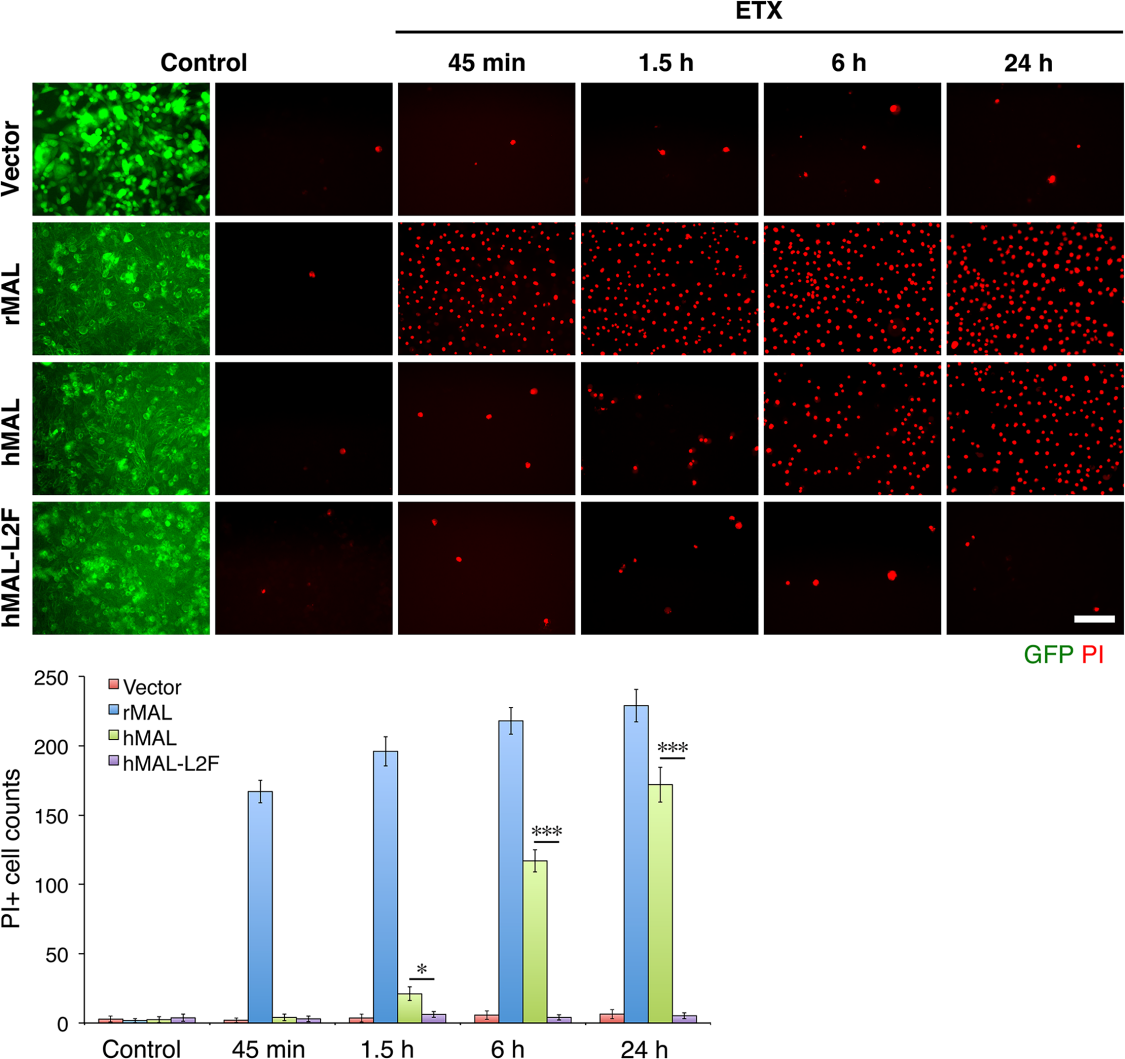
The second extracellular loop in MAL is critical to MAL-mediated ETX-cytoxicity. Representative images (top panels) and statistical analysis (bottom panel) show that MAL expression renders cells sensitive to ETX-induced cell death in rat MAL (rMAL)-expressing or human MAL(hMAL)-expressing CHO cells, whereas insertion of a FLAG segment in loop 2 of hMAL (hMAL-L2F) blocks hMAL-mediated ETX-cytotoxicity. CHO cells were treated with PBS (control) or 50 nM ETX and fixed at the indicated time points. 2 μg/ml PI (red) were included in the culture medium to monitor cell death. Similar levels and patterns of GFP expression (green) in untreated (control) hMAL and hMAL-L2F cells suggest blockade of ETX-toxicity in the latter cells is a consequence of structural alteration resulting from Flag insertion in loop 2. Scale bar represents 100 μm. Data are means and SD from a single experiment and are representative of three independent triplicate experiments.

Having shown that the second extracellular loop of MAL is required for ETX binding, we next tested the sensitivity of the cells expressing hMAL-L2F using PI exclusion as an assessment of cell death. As previously noted, cells expressing rMAL were more sensitive to ETX in cell death assays than those expressing hMAL. Cells expressing hMAL-L2F are completely insensitive to ETX and thus behave like GFP vector control cells (**Fig 6**). Even after 24 hours of exposure to 50 nM ETX, hMAL-L2F expressing CHO cells show no evidence of cell death (**Fig 6**). By contrast, the wild-type hMAL-expressing cells exhibit significant cell death after 6 hours of ETX exposure. Since the hMAL-L2F mutant protein is correctly targeted and oriented within the plasma membrane, these results support a specific requirement for ECL2 in ETX binding and cytotoxicity.

**Fig 7:**
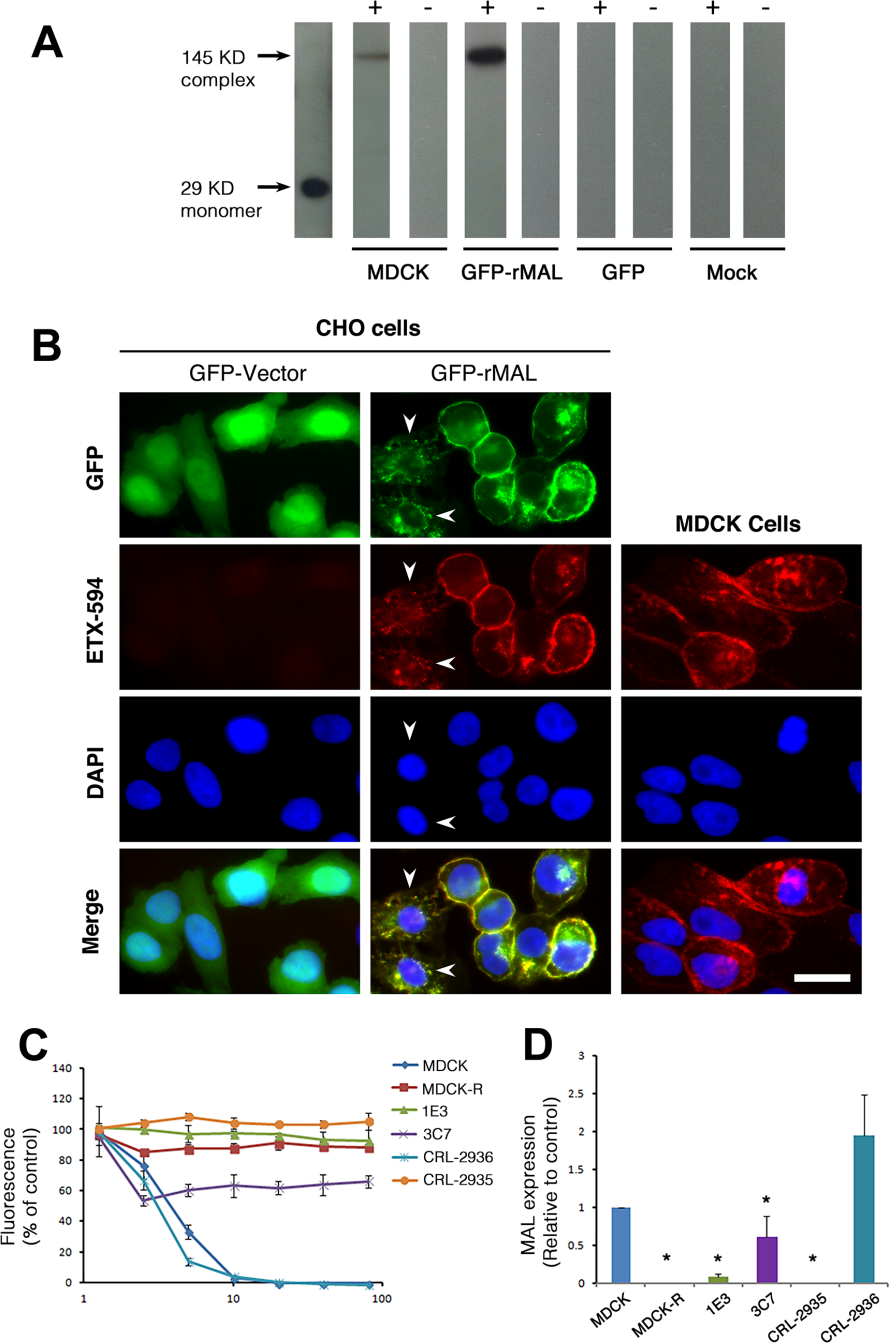
ETX pore formation, subcellular localization, and toxicity in cells are closely associated with MAL expression. (**A**) Formation of ETX pore complexes in MDCK cells and rMAL-expressing CHO cells. Western-blots of cell extracts treated with active ETX (+) or control buffer (-) for 1 hour at 37 C°. Detergent resistant membranes were isolated from ETX or control treated cells and solubilized in Lamelli buffer under non-reducing conditions without heating. Treatment of CHO^GFP-rMAL^ cells with active ETX leads to formation oligomeric complexes (GFP-rMAL) that do not form in ETX treated CHO^GFP^ cells (GFP) or mock transfected CHO cells (Mock). The relative molecular weight (MW) of the oligomeric complex formed in CHO^GFP-rMAL^ cells is similar to the size of the ETX oligomeric complex formed with ETX treatment of MDCK cells (MDCK), shown at 145 kDa on the blots. ETX monomer runs at 29 kDa. (**B**) ETX co-localizes with MAL on the cell membrane, and binds to MAL-expressing CHO cells and MDCK cells in a similar pattern. CHO cells stably expressing GFP-vector or GFP-rMAL fusion protein (GFP-rMAL) and MDCK cells were incubated with 25 nM ETX-594 conjugate (red) for 30 minutes before fixation for fluorescence analysis. DAPI (blue) were counterstained to reveal cell nuclei. Arrowheads point to CHO cells displaying co-localized ETX-MAL intracellular vesicles indicative of ongoing internalization. Scale bar represent 20 μm. (**C** and **D**) ETX sensitivity correlates with MAL expression levels in MDCK cells. Cell viability assays (C) show that various MDCK cell lines or cell culture manipulations exhibit different sensitivity to ETX-induced cell death. MDCK (CCL-34) cells were treated with a dose of ETX sufficient to kill >95% of the monolayer and individual surviving cells were recovered as a pool (MDCK-R) or as individual clonal populations (1E3 and 3C7). In addition, two additional MDCK cell derivatives (CRL-2935 and CRL-2936) were acquired from ATCC. Sensitivity to ETX was determined by incubating cell monolayers with serial dilutions of purified ETX. Quantitative RT-PCR analysis (D) shows that ETX-sensitive cells exhibit significantly higher levels of MAL expression compared to the cell lines resistant to ETX treatment. Data are from a single experiment and are representative of four (A), three (B), or at least three (C and D) independent triplicate experiments. Data shown are means and SD. Comparisons between MDCK and the other cell lines were performed by ANOVA followed by Dunnett’s *post hoc* test. * P<0.05.

MDCK cells are possibly the most thoroughly studied ETX-sensitive cell line, and we therefore wanted to compare the binding and sensitivity of ETX between MDCK and MAL expressing CHO cells. ETX is a 29 KDa protein that heptamerizes [46, 48, 73] after binding to its cell surface specific receptor, generating an asymmetric pore leading to ATP depletion and loss of ion and water homeostasis [74]. To determine if MAL expression in CHO cells is required for ETX heptamerization, we treated CHO^GFP^, CHO^GFP-rMAL^ and MDCK cells with ETX and performed Western-blot analysis of cell lysates for oligomerized ETX. As anticipated, treatment of MDCK and CHO^GFP-rMAL^ cells with ETX result in formation of an approximate 145 kDa complex (**Fig 7A**) consistent with a heptameric pore [46, 73]. Both MDCK and CHO^GFP-rMAL^ cells show similar localization patterns of ETX binding to the cell surface (**Fig 7B**). We next determined the relationship between MAL expression level and ETX sensitivity by analyzing various MDCK cell lines either sensitive or resistant to ETX (**Fig 7C** and **7D**). MDCK (CCL-34) cells were treated with a dose of ETX sufficient to kill >95% of the monolayer and individual surviving cells were recovered as a pool (MDCK-R) or as individual clonal populations (1E3 and 3C7). In addition, two additional MDCK cell derivatives (CRL-2935 and CRL-2936) were acquired from ATCC. Sensitivity to ETX was determined by incubating cell monolayers with serial dilutions of purified ETX (**Fig 7C**). In all cases ETX-sensitive MDCK cells express MAL and ETX-resistant MDCK cells show no expression or negligible levels of MAL (**Fig 7D**).

**Fig 8:**
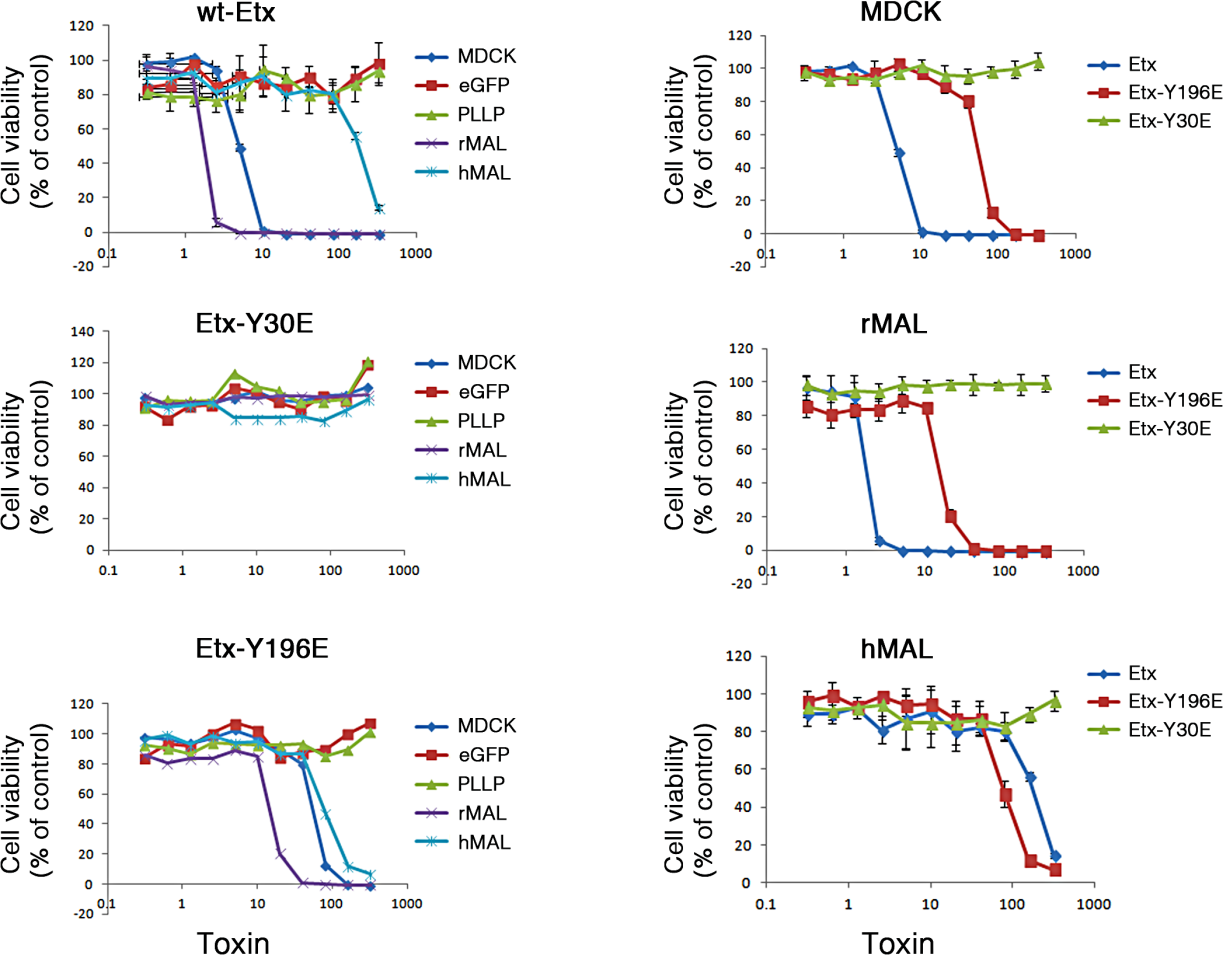
Specific aromatic residues within domain 1 of ETX are required for toxin activity in MAL-expressing CHO cells. The plots on the left show the cytotoxicity results on MDCK cells, CHO cells expressing rMAL or hMAL, and the two control CHO lines (GFP and the tetraspan PLLP). Cells expressing rMAL and MDCK cells show similar sensitivity to ETX whereas the cells expressing hMAL are less sensitive. The Y30E mutant eliminates ETX toxicity in all cell lines, while the Y196E mutant significantly attenuates the toxin activity in MDCK cells and rMAL cells. The plots on the right compare the same data by cell line rather than by toxin. On both MDCK and on cells expressing rMAL, about 10-fold greater amounts of Y196E versus wt-ETX are required to kill cells. In contrast, with cells expressing hMAL, there is little difference between the wt-ETX and the Etx-Y196E mutant. Data are means and SD from a single experiment and are representative of three independent triplicate experiments.

Previous studies have shown that a group of closely spaced aromatic amino acid residues within domain 1 of ETX are necessary for binding and toxicity to MDCK cells [44, 75, 76]. Specifically, glutamate substitutions at Y29, Y30, Y36 or F199 each result in loss of ETX binding, pore formation and cytotoxicity [44]. At Y196, also within domain 1, glutamate substitution result in significantly reduced ETX activity [44]. Here, we tested whether these ETX mutations similarly impact ETX toxicity in MAL-expressing CHO cells. Wild-type ETX (wt-Etx), mutant toxin containing the Y30E substitution (Etx-Y30E), and mutant toxin containing the Y196E substitution (Etx-Y196E) were examined, and rMAL- or hMAL-expressing CHO cells were compared to MDCK cells in cytotoxicity assays (**Fig 8**). As controls, we included CHO cells expressing the irrelevant tetraspan PLLP [77] as well as GFP vector alone. As expected, wild-type toxin induces robust cell death in MDCK cells and in CHO cells expressing rMAL or hMAL. rMAL-expressing CHO cells are more sensitive than MDCK cells, which exhibit greater sensitivity than hMAL-expressing CHO cells. Consistent with published reports [44, 75, 76], Y30 mutation (Etx-Y30E) completely abolishes ETX-induced cell death while Y196 mutation (Etx-Y196E) significantly reduces ETX-toxicity in MDCK cells. Remarkably, the effects of these ETX mutations on MDCK cells are essentially reproduced in MAL-expressing CHO cells. Y30 mutation (Etx-Y30E) renders ETX ineffective in both rMAL- and hMAL-expressing CHO cells. Intriguingly, while Etx-Y196E attenuates ETX effect approximately 10 folds in rMAL-expressing CHO cells compared to Wt-Etx, this mutant toxin slightly increases ETX toxicity in hMAL-expressing CHO cells. PLLP-expressing CHO cells are completely insensitive to wild-type ETX, indicating the specificity of MAL for toxin activity. These findings demonstrate that MAL expression alone is not only necessary to impart ETX toxicity in insensitive naïve CHO cells but is also able to confer differential response to ETX mutants. Moreover, our data suggest that ETX-MAL interaction involves the aromatic side chains in domain 1 of ETX and the second extracellular domain of MAL.

We have so far provided evidence that introduction of MAL into ETX-resistant cells renders them capable of binding to toxin and sensitive to its effects. We further showed that insertion of the FLAG epitope into ECL2 results in loss of ETX toxicity. To elaborate on the loss-of-function strategy, we made use of the mice deficient in MAL (MAL-/-) [55], which exhibit no overt physical or behavioral abnormalities but do have a defect in the maintenance of CNS paranodal myelin loop structure [55]. We first investigated the behavioral responses of animals injected with ETX using a detailed scoring system. Adult mice were injected via the tail vein with 5 ng of ETX per gram of body weight and then placed under continuous observation for an hour. The mean time for onset of symptoms following ETX administration is 11.8 minutes for wild-type mice (**Table 1**), and all of the wild-type mice undergone ETX injection progressed rapidly to a moribund state requiring euthanasia. None of the MAL-/- mice injected with ETX developed symptoms and they remain healthy throughout the next 48 hours after which they were routinely sacrificed. Neither wild-type nor MAL-/- mice in the untreated control groups developed behavioral symptoms. In a second experiment, mice were injected with 50 ng of ETX and videotaped. MAL-/-mice were completely resistant to ETX, whereas wild-type mice rapidly became obtunded (**S1 Movie**).

**Table 1:** Symptom-onset time following ETX administration in mice.

Genotype	Treatment	Number of mice	Symptom-onset time
MAL+/+	Control	5	None observed
MAL+/+	ETX	8	11.8 ± 2.9 min
MAL-/-	Control	4	None observed
MAL-/-	ETX	5	None observed

2–5 month-old wild-type (MAL*+/+)* or MAL-Knockout (MAL*-/-)* mice were untreated (control) or injected via the tail vein with ETX at the dose of 5 ng/gram of body weight. Time until symptoms was recorded for the next hour. Data are means and SD from a single experiment and are representative of two independent experiments

**Fig 9:**
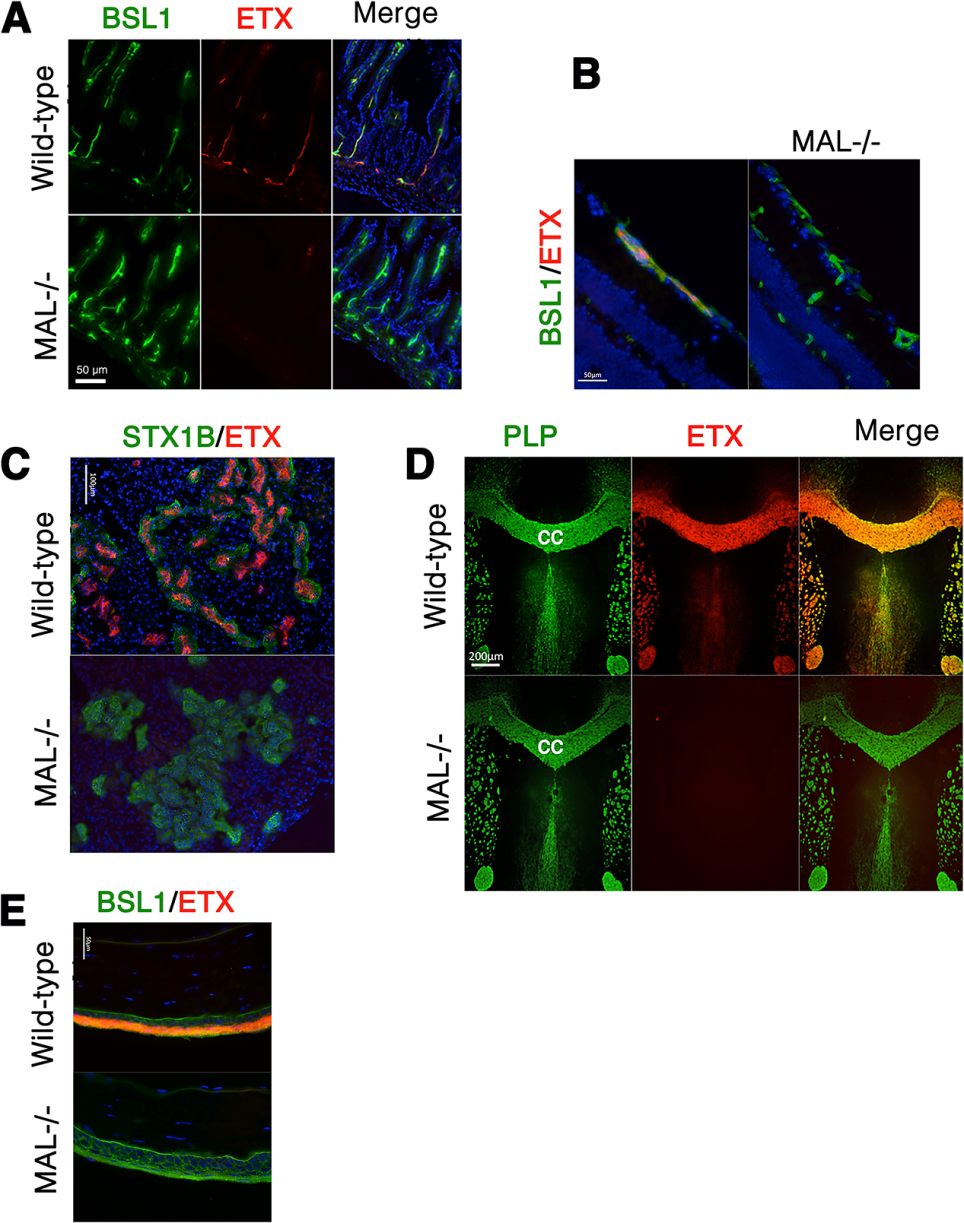
MAL is necessary for ETX binding *in vivo*. Alexa-594-conjugated-ETX binds to tissue from wild-type mice but not MAL-/- mice. **(A)** Mice were injected intravenously with Alexa-594-conjugated-ETX (red) and BSL1 (green), a pan endothelial cell marker, followed by PBS perfusion. ETX (red) binds to vascular endothelium of the small intestine in wild-type mice but not MAL-/- mice. To evaluate ETX binding to retinal vessels **(B)**, kidney **(C)**, CNS white matter **(D)**, and squamous epithelium of the eye sclera (**E**), tissue sections were probed with Alexa-594-conjugated-ETX (red). ETX binds to tissue from wild-type mice but not MAL-/- mice. BSL1 (green) was stained in (A), (B), and (E) to identify endothelial cells. Shiga toxin (STX1B)-488 (green) was used to identify kidney tissue in (C). The myelin marker PLP (green) was stained to identify myelin structures in the brain in (D). Note that on the brain section (D), ETX shows nearly complete overlap with myelin stains. The corpus callosum (cc), which is the major white matter tract in the brain, is marked. DAPI (blue) was counterstained to identify cell nuclei. Data are representative of at least three independent experiments.

We next examined binding of ETX to tissues from wild-type and MAL-/- mice [55]. In wild-type mouse tissues examined, Alexa-594-labeled ETX stains gut microvasculature, retinal microvessels, renal tubules, CNS myelin, and squamous epithelium of the eye sclera (**Fig 9**). However, ETX fails to stain these same structures from MAL-/- mice (**Fig 9**). Staining of markers for CNS myelin (PLP), microvasculature and eye sclera epithelium (BSL1), and renal tubules (Shiga toxin 1 beta) show no difference between wild-type and knockout mice. These results indicate that MAL loss-of-function abrogates specific ETX binding *in vivo*.

## Discussion

### Is MAL the ETX receptor?

We have shown that introduction of the MAL gene imparts ETX sensitivity to previously insensitive CHO cells, and that the characteristic responses of MDCK cells to ETX are shared by MAL-expressing CHO cells. These cellular responses include cell surface binding of ETX, ETX oligomerization and pore assembly, differential response to ETX mutant proteins according to the nature of the mutations [43, 44, 76, 78], and cell death. Such remarkable consistencies led us to speculate that MAL is either 1) the bona fide ETX receptor and interacts directly with ETX via protein-protein interactions or 2) part of the ETX receptor complex and plays a role in the sorting, chaperoning, and/or recruitment of a novel direct binding partner of ETX.

We employed several different experimental approaches to determine if ETX interacts directly with MAL, however, the results of these experiments were inconclusive. Numerous variations on traditional co-immunoprecipitation (co-IP) and far-Western blotting experiments altering antibodies, salt concentrations and detergents yielded inconsistent results. One possible explanation for these inconsistences is that transmembrane proteins containing multiple hydrophobic domains, as is the case of MAL, often possess different tertiary structures and binding affinities when in solution relative to those occurring within a lipid bilayer [79]. Such changes might confound solution-based analysis such as co-IP and far-Western detection or even render physical interaction between ETX and MAL impossible in these conditions. We also obtained negative results from far-Western blotting and fluorescence resonance energy transfer (FRET) experiments. While FRET analysis was performed under physiologic conditions with MAL in its native conformation within a lipid bilayer, molecular orientation and physical distance between the relevant fluorophores on ETX and MAL could easily preclude FRET detection. Investigation using other advanced methods for detecting protein-protein interactions, including co-IP/protein mass fingerprinting and surface plasmon resonance biosensor assays are currently underway. Our results thus far do not distinguish between a function of MAL in direct binding of ETX versus a role of MAL in recruiting or organizing the ETX receptor complex.

Although we have been unable to determine if MAL interacts directly with ETX, theoretical modeling suggests MAL may have the structural requirements to facilitate direct protein-to-protein interaction. The primary structure of the extracellular loop 2 (ECL2) for MAL reveals a Q to F substitution between human and rat respectively at position 114, a change from the highly polar side chain of glutamine to the non-polar side chain of phenylalanine. Considering the functional significance of aromatic side chains in the receptor-binding domain of ETX [44], the aromatic residue in ECL2 for rMAL may contribute to additional aromatic stacking interactions with residues in the receptor-binding domain of ETX [44]. If MAL is the binding receptor for ETX, then the second ECL appears to play an essential role. Insertion of the FLAG epitope into ECL2 leads to abrogation of both ETX binding and cytotoxicity while retaining normal localization of MAL in the plasma membrane. Computer-aided analysis predicts that the ECL2 FLAG sequence insertion changes structural dynamics of ECL2- particularly in the aspect of protein surface accessibility. This, if experimentally proved, would suggest a central role for ECL2 structural integrity in ETX binding and toxicity. Interestingly, previous studies using the same construct show FLAG insertion in ECL2 neither alters the incorporation of MAL into glycolipid-enriched membrane microdomains and lipid rafts [80], nor influences MAL trafficking between the cell membrane and the trans-Golgi network [56]. That the FLAG insertion in ECL2 results in loss of ETX binding and toxicity thus favors, but does not prove, a direct role of MAL in transmitting ETX activity into target cells.

Despite MAL’s structural possibilities as the ETX receptor and the requirement of MAL for both ETX binding and toxicity, it is possible that MAL is not the direct binding receptor for ETX. MAL could be functioning in ETX binding either as a co-receptor, part of a multmeric protein complex, or as a membrane lipid micro-domain organizer necessary for assembly of the receptor complex, without directly binding to ETX. The lethal effects of *C*. *perfringens* ETX require first, binding of ETX monomers to a specific cell surface receptor; second, oligomerization into a prepore complex; and third insertion of the stable pore into the plasma membrane culminating in the free passage of ions and water and thus loss of electrochemical gradients essential for cell viability [46–48, 73]. MAL could play a role in any of these required steps without directly interacting with ETX. Indeed, MAL is known to localize to the apical surface or within specialized microdomains of particular polarized cells [49, 53, 56–60, 63, 80–83]. Localization of MAL on cell surface and MAL’s role in protein sorting provide the ideal localization, microenvironment and cellular machinery for ETX to target and damage cells [54, 55, 58, 64, 82, 84].

Previous studies show that HAVCR1 is required, at least partially, for ETX-mediated cytotoxicity [37, 44]. HAVCR1 increases the sensitivity of cells to ETX if they are already sensitive to the toxin. However, HAVCR1 expression alone is insufficient to confer ETX sensitivity upon cell lines resistant to the ETX effect. Also, both ETX binding and ETX-mediated cell death occur in the absence of HAVCR1. Moreover, ETX binding with HAVCR1 on cell surface has not been demonstrated [37]. In contrast, cells insensitive to ETX become completely sensitive when MAL expression is induced. In addition, ETX binds to the MAL-expressing cell membrane surface and becomes internalized in a time-dependent fashion. Thus, MAL seems to play a more central role than HAVCR1 in the context of ETX binding and toxicity. It would be interesting to determine whether MAL is physically and/or functionally linked to HAVCR1 in this context.

Taken together, we have identified, for the first time, a protein that is required for both binding and activity of ETX to cells. These results provide novel insights on how this toxin interacts with host cells. Since ETX is responsible for a devastating disease in livestock and may be a possible environmental factor contributing to the initiation of new MS lesion formation, understanding the molecular basis of toxin binding to cells and cytotoxicity opens investigation into new treatment strategies.

### Does MAL structure and function make sense for enhancing ETX pore formation?

The normal physiological function of MAL in apical sorting of proteins to specialized lipid rafts affords the ideal context for a pore-forming toxin. ETX oligomerization requires close proximity of monomers to facilitate interaction between amphipathic residues within domain 3 of the active toxin [43]. Highly concentrated MAL within lipid rafts favors these necessary protein-protein interactions by bringing MAL-bound toxin monomers within binding range of one another [54, 55, 58, 64, 82, 84]. Intriguingly, MAL itself has been shown to self-oligomerize [64], which may further support ETX oligomerization and pore formation. The same membrane biophysical characteristics achieved within the lipid raft microenvironment that enhance endosome formation also appear to enhance pore formation. Lipids that favor spontaneous negative curvature in bilayers such as cholesterol, diacylglcerol, long-chain unsaturated fatty acids, glycosphingolipids and phosphatidylethanolamine are enriched in lipid rafts and have been shown to enhance ETX pore formation [34, 36]. Caveolins 1 and 2, essential scaffolding proteins localized to lipid rafts, promote formation of caveolae and are necessary for oligomerization of ETX and pore formation [85]. In essence, the MAL microenvironment is well suited for a pore-forming toxin that requires oligomerization.

### What accounts for the variability in ETX pathogenicity between species?

Why are the pathological effects of ETX so variable between species? In part, this occurs due to known differences in the kinetics of bacterial growth within the gastrointestinal tracts of species with fermenting 4-chamber stomachs (ruminant) and the linear gut of the rodent or human [3, 7, 86–98]. A second level of difference explaining the distinct pathologic effects of ETX between species is receptor affinity. The binding receptor for ETX remains undefined. In this report we have shown that MAL is required for both ETX binding to cells and for cellular toxicity. Interestingly, the binding affinity and the susceptibility of cells to cytotoxic effects of ETX vary dramatically between rodent and human. Cells expressing human MAL bind ETX with approximately 1/10 the affinity of cells expressing rat MAL, and cells expressing zebrafish MAL do not bind ETX at all. The differences in affinity and cytotoxicity of ETX for cells expressing different MAL orthologues offers an additional level of complexity for the remarkable differences in toxin mediated pathologies between species. It will be of interest to test the relative binding affinity of ETX to MAL from a wide range of species.

## Materials and Methods

### Ethics statement

All animal work was conducted according to federal guidelines and approved by the Weill Cornell Medical College Institutional Animal Care and Use Committee.

### Generation of MAL constructs

The pβ-actin-EGFP-rat MAL (rMAL) and human MAL (hMAL) constructs and hMAL harboring a FLAG sequence insertion in its second extracellular loop (hMAL-L2F) have been previously published [56, 58]. hMAL and hMAL-L2F were subcloned and inserted into the N-terminal of GFP gene in the pβ-actin-EGFP vector. hMAL with a FLAG insertion in the first extracellular loop (hMAL-L1F) was synthesized with RsrII/XbaI restriction sites, and purchased from Genewiz, Inc. A plasmid encoding zebrafish MAL was purchased from ATCC, subcloned and inserted into the pβ-actin-EGFP vector. The pEGFP-C1 vector was purchased from Addgene and used as the GFP control. All plasmids were amplified and purified (Qiagen) in One Shot Top 10 *Escherichia coli* (Life Technologies). Sequence analysis and verification was performed (Macrogen USA).

### ETX

ETX and pro-ETX are provided by BEI at a minimum > 95% purity. Each lot was provided with a certificate of analysis including a Western-blot, which showed a dominant band at ~30 kDa. In some lots, a faint low molecular weight band was also observed. Importantly, use of an ETX specific neutralizing antibody completely abrogated the cell death-inducing activity of the reagent underscoring its purity.

### Fluorophore labeling of ETX/pro-ETX

His-tagged **ε**-prototoxin was procured from BEI Resources. One mg was fluorescently labeled using Alexa Fluor 594 Protein Labeling Kit (Life Technologies) and 1 mg was labeled using Alexa Fluor 647 Protein Labeling Kit (Life Technologies) as per manufacturer's instructions. His tagged Shiga toxin 1β was procured for BEI Resources and fluorescently labeled using Alexa Fluor 488 Protein Labeling Kit (Life Technologies).

### Expression and purification of recombinant ε -prototoxin

Expression and purification of recombinant wild-type and mutant ε-prototoxins were performed as described previously [44, 72]. Purified ε-prototoxins were treated with trypsin-coated agarose beads and conversion of the prototoxin to active toxin was assessed based on SDS-PAGE and immunoblotting with anti-ETX antibodies [37].

### Cell culture and transfection

Chinese hamster ovary (CHO) cells (ATCC Cat#CCL-61) were grown in Dulbecco's Modified Eagle's Medium/Ham's F12 medium (Life Technologies) with 10% heat-inactivated fetal bovine serum, 50 units/ml penicillin and 50 μg/ml streptomycin. CHO cells were transfected with the indicated expression plasmids using Turbofect (Fermentas) according to the manufacturer’s instructions. For stable cells, clumped cells were collected after transfection and growth in media containing G418 400 μg per ml for three weeks. Cells were then analyzed under a fluorescence microscope for GFP expression. Homogenous GFP expressing cells were selected using a Becton-Dickinson Vantage cell sorter. Colonies derived from single cells were viable and expanded to generate frozen stocks. MDCK cells were routinely cultured in Minimum Essential Medium (MEM) supplemented with glutamax (Life Technologies) and 10% FetalClone III serum (HyClone) and incubated at 37°C in 5% CO2.

### Cell viability assays

#### PrestoBlue assay

Stably transfected rat GFP-MAL (CHO^GFP-rMAL^) and GFP (CHO^GFP^) cells were grown in 384-well plates and incubated with ETX overnight. PrestoBlue Reagent (Life Technologies), which is quickly reduced by metabolically active cells to a red fluorescent metabolite, was added to each well using a multi-drop liquid dispenser. Fluorescence intensity was measured with a microtiter plate reader (Perkin-Elmer Envision). For cytotoxicity analyses of MDCK cells, cells were plated in Leibovitz L-15 medium supplemented with 10% fetal bovine serum at a density of 5×10^3^ cells per well in 384-well plates [66]. ETX was added and the cells were incubated at 37°C overnight. Cytotoxicity was determined by treating cells with resazurin (CellTiter Blue, Promega) at 37°C for 4 hours. Fluorescence was measured at 590 nm following excitation at 560 nm using a BioTek FLx800 plate reader. Results were normalized to the fluorescent signal from cells incubated in the absence of toxin (100%) and in 0.1% Triton X-100 (0%).

#### Propidium iodide (PI) uptake assay

Cell membrane permeability was assessed by the ability of cells to exclude the DNA-binding fluorescent dye PI. Specifically, CHO cells stably expressing GFP, GFP-rMAL (rMAL), GFP-hMAL (hMAL), or GFP-hMAL-L2f were treated in triplicate wells for each condition indicated in the experiments. Live images of randomly chosen fields in each well were acquired under an inverted fluorescence microscope (Nikon) equipped with a CCD camera (Carl Zeiss), and were then imported into ImageJ64 (NIH) in 8-bit grey format. For quantification of PI-positive cells, the images were converted into binary images by applying the same threshold value to all images collected from the same experiment. Analyze Particles function was selected to automatically count the particle numbers and to analyze particle properties, such as size shape, and distribution patterns. Data were exported into Excel (Microsoft) and statistical significance was assessed by two-tailed student’s *t*-test. In the experiment indicated, the toxin dose required to kill 50% of the cells (IC_50_) was determined by nonlinear regression analysis in Prism (GraphPad Software).

### Flow cytometry

Transfected cells were removed from the tissue culture substrate by incubating with citric saline (135μM potassium chloride, 15μM sodium citrate dissolved in sterile water) for 5 minutes at 37°C. Cells were washed off the plate and then triturated into single cell suspensions. Cells were washed 3 times with DMEM without phenol red (Life Technologies), containing 10% fetal calf serum, 50 U/ml penicillin, and 50mg/ml streptomycin. Cells were diluted to 5x10^5^/ml and incubated with 20 nM Alexa 647-labeled ETX for 1 hour at 37°C. Labeled cells were washed 3 times in DMEM lacking phenol red, and read on a AccuriC6 flow cytometer (BD Biosciences).

### Fluorescence microscopy

Cells stably transfected with rat MAL (CHO-MAL) or GFP (CHO-GFP) were plated on glass bottom dishes (Mattek) at a density of 6x10^4^ cells/ml, and allowed to grow overnight. Cells were subsequently washed and fixed with 4% PFA for 10 minutes at room temperature (RT). Cells were washed and incubated with Alexa 594 labeled ETX (50nM) for 1 hour at RT. After incubation, the cells were washed 3 times in PBS and Hoechst (1μg/ml) was added for visualization of nuclei. Stained cells were visualized by confocal microscopy at the Rockefeller University Bio-Imaging Facility.

### Analyses of ETX binding and ETX-MAL co-localization

CHO cells stably expressing GFP, GFP-rMAL, GFP-hMAL or GFP-hMAL-L2F cultured in triplicated wells were incubated with ALexa 594-labeled ETX (ETX-594) for an hour. In some experiments, MDCK cells were incubated with ETX-594 for 30 minutes. Cells were fixed then with 4% PFA for 10 minutes at RT and were subsequently mounted onto slides in VECTASHIELD mounting medium containing DAPI (Vector Laboratories). Digital images were acquired under Axiskop2 fluorescence microscope (Carl Zeiss) coupled with a SPOT cooled camera (Diagnostic Instruments) and were imported into ImageJ64 (NIH) in 8-bit grey format. Images for GFP-expression and ETX-binding intensity analyses were processed in separate channels and converted into binary images by applying the same respective threshold values. Measurement was automatically performed on the binary images for mean and integrated fluorescent density. Data were exported into Excel (Microsoft) for statistical analysis.

### FLAG expression analysis

CHO cells stably expressing GFP-hMAL or GF-hMAL-L2F were incubated with the anti-FLAG antibody (Sigma) at 37°C for 10 minutes, followed by two quick washes with Hank's Balanced Salt Solution (HBSS, Life Technologies). Cells were fixed with 4% PFA at RT for 10 minutes and then mounted onto slides in VECTASHIELD mounting medium with DAPI (Vector Laboratories). Images were acquired under Axiskop2 fluorescence microscope (Carl Zeiss) coupled with a SPOT cooled camera (Diagnostic Instruments) and processed in Photoshop (Adobe).

### MAL expression in cell lines

Total RNA was isolated from cells with TRIZol reagent (Life Technologies). Complementary DNA (cDNA) was prepared from 2 μg total RNA using iScript cDNA synthesis kit (Bio-Rad). Quantitative real-time PCR was performed using a MAL-specific primer set from Applied Biosystems and a StepOne Plus instrument (Applied Biosystems). A primer set specific to 18S rRNA was used as control. Results were analyzed by comparing CT values [99].

### Western-blot analysis of the ETX complex

MDCK, CHO^GFP-rMAL^, CHO^GFP^ and mock transfected CHO cells (CHO^mock^) cells were grown in 6- well plates (Costar) until confluence. Activated ETX (60nM) or vehicle control was administered to cells and allowed to incubate for 1 hour at 37 C°. Cells were detached from plate following trypsin digestion. Cells were collected, washed 3 times in PBS and re-suspended in PBS containing 0.2% SDS. Harvested cells were then triturated with a serological pipette followed by trituration with an insulin syringe until DNA was sufficiently sheared. ETX complex containing detergent resistant membranes was isolated by centrifugation at 14, 000G for 5 minutes and collecting the pellet. The pellet was washed 3 times in PBS and re-suspended in 100 ul PBS. An equal volume of 2 X Lamelli sample buffer (Bio-rad) was added to each sample for SDS page electrophoresis. Proteints were transferred to an Immobilon-P membrane (Millipore) and probed with a custom rabbit anti-ETX antibody generated against the N terminal of ETX sequence KASYDNVDTLIEKGR (Pacific Immunology). HRP conjugated donkey anti-Rabbit IgG (Jackson Immunoresearch) was used to visualize the ETX complex.

### Immunofluorescence analysis with mouse tissues

#### Brain

Fresh frozen tissue sections were fixed in 4% PFA for 10 minutes at RT, permeabilized in a 1% sodium cholate, 1% BSA, 10% donkey serum, PBST solution overnight at 4°C^16^. Sections were then incubated with rabbit anti-PLP (ThermoScientific) at 1:1000 overnight at 4 C°. Following three washes with PBS, sections were then incubated with donkey anti-rabbit Alexa 488 (Jackson ImmunoResearch) at 1:1000, and Alexa 594 labeled His-tagged protoxin (50nM) for 2 hours at RT.

#### Retina/eye

Fresh frozen tissue sections were incubated with BSLI (Vector Labs) 1:200, and Alexa 594 labeled His-tagged protoxin (50 nM) for 1 hr at RT. After three 5-minute washes in PBS, stained sections were post fixed in 4% PFA for 10 minutes at RT.

#### Kidney

Fresh frozen tissue sections were fixed in 4% PFA for 10 minutes at room temperature, washed and incubated with Alexa 488 labeled His-tagged Shiga toxin 1β (200 nM) and Alexa 594 labeled His-tagged protoxin (50 nM) for 1 hour RT.

The above stained tissues were washed 3 times in PBS, mounted and imaged under Axiskop2 fluorescence microscope (Carl Zeiss) coupled with a SPOT cooled camera (Diagnostic Instruments.

#### Intestines

Anesthetized wild-type and MAL-/- mice were injected intravenously via tail vein injection with 1ug Alexaflour 594 labeled active ETX and 1.6 μl FITC-conjugated BLS1 (Vector Laboratories) per gram of mouse. After ten minutes, mice were euthanized by cardiac perfusion with PBS. Tissue was removed and fresh frozen in OTC.

### Mouse lines

MAL-deficient mice (MAL-/-) were generated by replacing the first exon of the *MAL* gene with the *lacZ* gene sequence [55]. MAL-/- mice were backcrossed with C57 / Bl6 mice for more than six generations. MAL-deficient mice bred normally and had a normal life span.

### ETX toxicity in mice

#### Animal handling

All animal work was conducted under an approved WCMC animal protocol. Each mouse received ETX via intraperitoneal injection. Animals were injected once IP with the dose of ETX indicated or with vehicle. Behavior was monitored every 15 minutes for the first 2 hours and every half hour for the next six hours. If animals showed signs of severe neurological toxicity (see below) then they were sacrificed by a lethal injection of Ketamine/Xylazine (450 mg/kg Ketamine IP: 45 Xylazine mg/kg IP).

#### Scoring system and actions for humane care of animals

For all animals a medical record and a neurological scoring chart was kept. For neurological signs the scoring system used was the same as for experimental allergic encephalomyelitis experiments. The scoring system was as follows: 0: No abnormality. Action: Continued monitoring. 1: Initial signs but no paraparesis. This included clumsiness, incontinence, flaccid tail. Action: Moistened food and hydration packs were added to the cage bottom. Continued monitoring. 2: Mild paraparesis: Characterized by trouble initiating movement, but walking well once started. Action: Affected mice were fed with moistened food and gel hydration packs. Continued monitoring. 3: Moderate paraparesis: Characterized by an inability to move one or both hindlegs, noticeable gait disturbance, and possible atonic bladder. Action: Moistened food and hydration packs were placed on floor of cage. Expressed urinary bladder if necessary. Supplemental heat if necessary. 4: Moderate quadraparesis: Characterized by weakness in all limbs, associated gait disturbance and possible atonic bladder. Animals could still move and feed from moistened food or hydration packs. Action: Moistened food and hydration packs were placed on the cage floor. Animals in this state were monitored every 15–30 minutes. The urinary bladder was manually expressed if needed. Animals were provided an external means of heat, if needed (heat lamp, Safe and Warm, nestlets with additional bedding). If animals were unable to move or unable to feed, then they were euthanized immediately. 5: Moribund: Characterized by an inability to move even with prodding, inability to feed, or labored respirations. Action: Euthanized immediately.

### Data analysis and statistics

In most experiments, data were analyzed with Excel (Microsoft) and statistical significance was assessed by two-tailed student’s *t*-test. * p < 0.05; ** p < 0.01; *** p < 0.001. In MAL expression analysis, statistical different between samples were performed by ANOVA followed by Dunnett’s *post hoc* test. * P<0.05. In experiments determining ETX IC_50_ on vector-,rMAL- or, hMAL-expressing CHO cells, nonlinear regression analysis was performed in Prism (GraphPad Software).

### Accession numbers

Rat (Rattus norvegicus) myelin and lymphocyte mRMA/Mal NM_012798, Rat Mal protein NP_036930, Human (Homo sapiens) MAL mRNA NM_002371, Human MAL protein NP_002362, Dog (Canis lupus familiaris) Mal mRNA NM_001003253, Dog Mal protein NP_001003253, Zebrafish (Danio rerio) MAL-like protein mRMA NM_001017686, Zebrafish Mal-like protein NP_001017686, epsilon-toxin (Clostridium perfringens B strain ATCC 3626) gene ABDV01000020.1, epsilon-toxin protein EDT23265.1

## Supporting Information

S1 Fig Alexa-594-conjugated ETX retains toxicity in MAL-expressing CHO cells. CHO cells stably expressing rMAL were exposed to either purchased activated ETX from BEI (ETX, top row), Alexa-594 conjugated ε-ptotoxin (proETX-594, middle row), or trypsin-activated Alexa-594 conjugated pro-ETX (TA ETX-594, bottom row) for 1 hour and cell viability was assessed by PI (red) inclusion. Tyrpsin-activated Alexflour-594 conjugated pro-ETX exhibited similar cytotoxic activity as the purchased activated toxin from BEI, whereas protoxin has no effect on cell viability. Note that at the indicated concentrations, fluorescence from proETX-594 or TA ETX-594 binding is not detectable, allowing visualization of signals from PI-positive cells. Data are representative of at least three independent experiments.
(TIF)

S2 Fig Time-course of ETX-induced cell death in MAL-expressing cells. CHO cells stably expressing rMAL (CHO^GFP-rMAL^) or GFP-alone (CHO^GFP^) were treated with active ETX at the concentration of 500 pM for the times indicated and then incubated with PI. Live cultures were then examined for PI uptake (red) and GFP (green) by epifluorescence. CHO^GFP-MAL^ but not CHO^GFP^ cells become permeable to PI 2 hours after ETX treatment. CHO^GFP^ cells remain resistant to ETX pore formation even after a 24-hour incubation. Scale bar represents 125 μm. Data are representative of at least three independent experiments.
(TIF)

S1 Movie MAL is necessary for ETX toxicity *in vivo*. A wild-type mouse (foreground) that has received 50 ng of ETX via intraperitoneal injection (i.p.) lays obtunded. By contrast, a MAL-/- mouse (background) injected, i.p., with 50 ng of ETX remains fully alert and unaffected. Data are representative of four independent experiments.
(MOV)
