# Supplementary figures and images for: The Myelin and Lymphocyte Protein MAL Is Required for Binding and Activity of Clostridium perfringens ε-Toxin
Source: PLoS Pathog. 2015 May 20;11(5):e1004896. doi: 10.1371/journal.ppat.1004896 (PMC4439126; doi:10.1371/journal.ppat.1004896)

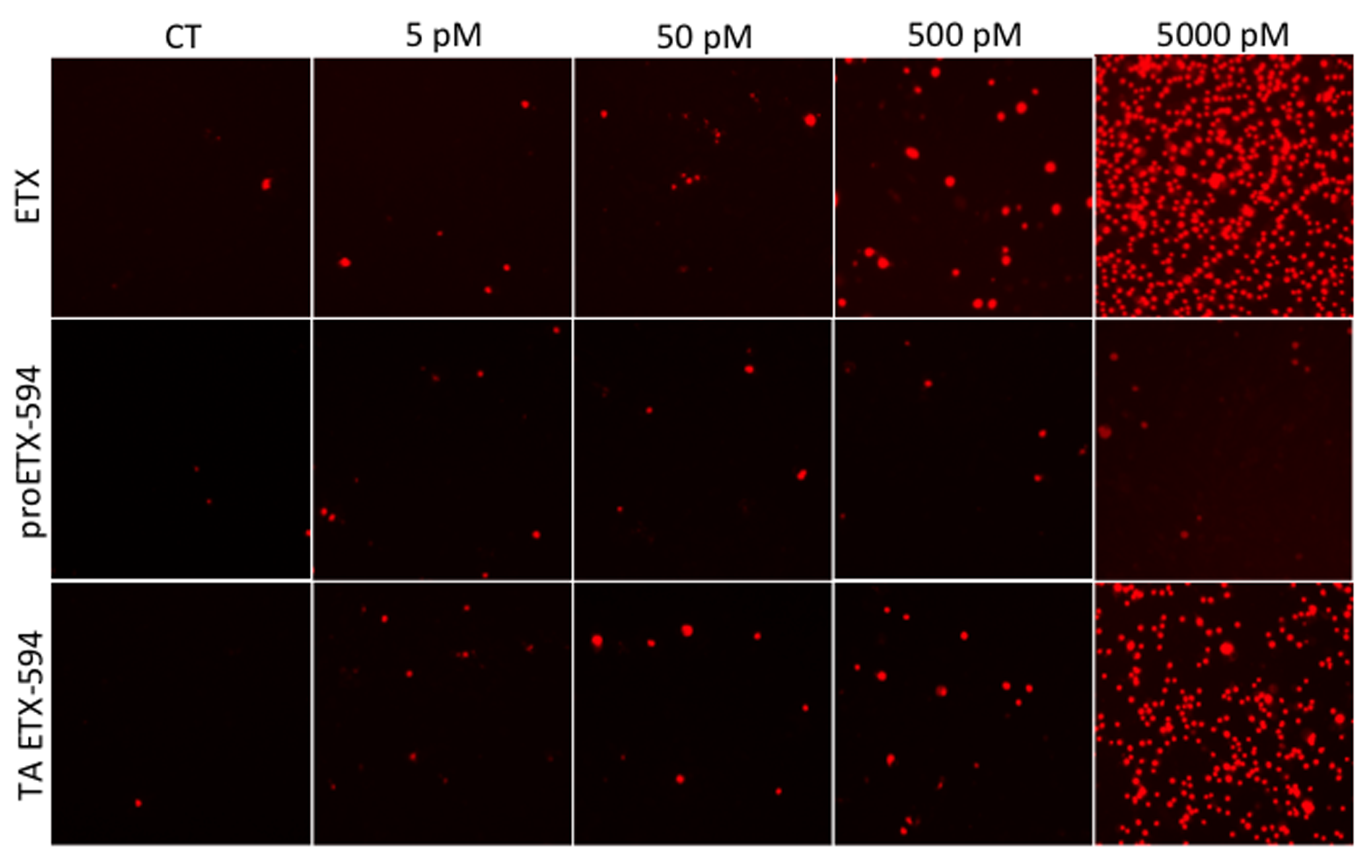

Supplement: S1 Fig — CHO cells stably expressing rMAL were exposed to either purchased activated ETX from BEI (ETX, top row), Alexa-594 conjugated ε-ptotoxin (proETX-594, middle row), or trypsin-activated Alexa-594 conjugated pro-ETX (TA ETX-594, bottom row) for 1 hour and cell viability was assessed by PI (red) inclusion. Tyrpsin-activated Alexflour-594 conjugated pro-ETX exhibited similar cytotoxic activity as the purchased activated toxin from BEI, whereas protoxin has no effect on cell viability. Note that at the indicated concentrations, fluorescence from proETX-594 or TA ETX-594 binding is not detectable, allowing visualization of signals from PI-positive cells. Data are representative of at least three independent experiments. (TIF) [file ppat.1004896.s001.tif]

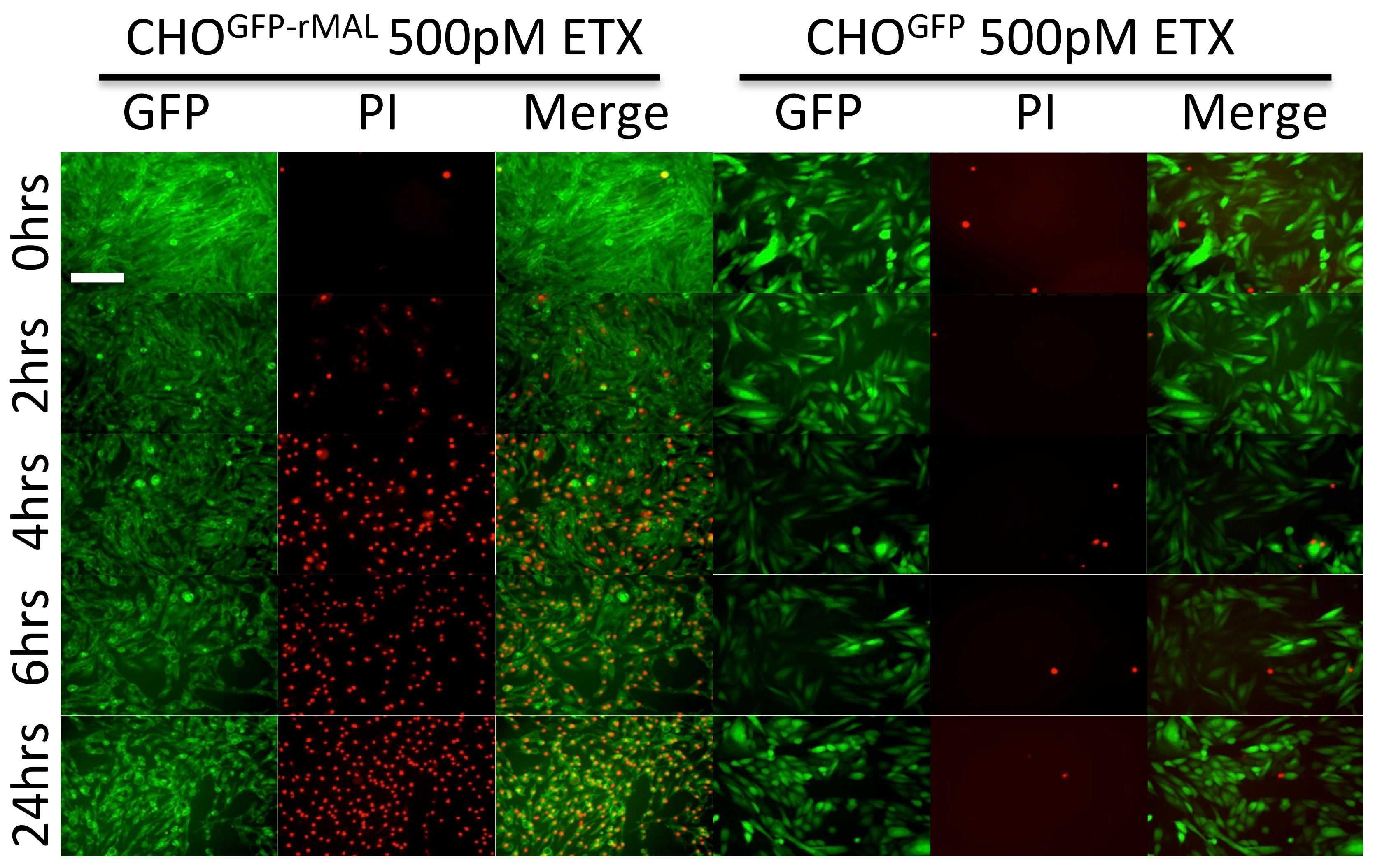

Supplement: S2 Fig — CHO cells stably expressing rMAL (CHOGFP-rMAL) or GFP-alone (CHOGFP) were treated with active ETX at the concentration of 500 pM for the times indicated and then incubated with PI. Live cultures were then examined for PI uptake (red) and GFP (green) by epifluorescence. CHOGFP-MAL but not CHOGFP cells become permeable to PI 2 hours after ETX treatment. CHOGFP cells remain resistant to ETX pore formation even after a 24-hour incubation. Scale bar represents 125 μm. Data are representative of at least three independent experiments. (TIF) [file ppat.1004896.s002.tif]
